# Discordant Clinical Outcomes in a Monozygotic Dichorionic-Diamniotic Twin Pregnancy with Probable Zika Virus Exposure. Case Report

**DOI:** 10.3390/tropicalmed5040188

**Published:** 2020-12-19

**Authors:** Marcela Mercado, Marcela Daza, Cynthia A. Moore, Diana Valencia, Angelica Rico, Diego A. Álvarez-Diaz, Aaron C. Brault, Kelly Fitzpatrick, Sarah B. Mulkey

**Affiliations:** 1 Division of Research in Public Health, National Institute of Health of Colombia, Bogota 110311, Colombia; mmercado@ins.gov.co (M.M.); arico@ins.gov.co (A.R.); dalvarezd@ins.gov.co (D.A.Á.-D.); 2 Vysnova Partners, Bethesda, MD 20785, USA; 3 National Center on Birth Defects and Developmental Disabilities, Centers for Disease Control and Prevention, Division of Birth Defects and Infant Disorders, Atlanta, GA 30333, USA; cam0@cdc.gov (C.A.M.); ile9@cdc.gov (D.V.); 4 National Center for Emerging and Zoonotic Infectious Diseases, Centers for Disease Control and Prevention, Division of Vector-Borne Diseases, Ft Collins, CO 80521, USA; zlu5@cdc.gov (A.C.B.); hwm8@cdc.gov (K.F.); 5 Prenatal Pediatrics Institute, Children’s National Hospital, Washington, DC 20310, USA; sbmulkey@childrensnational.org; 6 Departments of Pediatrics and Neurology, The George Washington University School of Medicine and Health Sciences, Washington, DC 20052, USA

**Keywords:** Zika virus, twins, brain anomalies, congenital infection, microcephaly, pregnancy

## Abstract

Prenatal exposure to Zika virus (ZIKV) is associated with congenital anomalies of the brain and the eye and neurodevelopmental sequelae. The spectrum of disease outcomes may relate to timing of infection as well as genetic and environmental factors. Congenital infections occurring in twin pregnancies can inform the clinical spectrum of these conditions and provide unique information regarding timing of infection and in utero environment with disease pathophysiology. Herein, we report a monozygotic dichorionic-diamniotic twin pregnancy with probable prenatal ZIKV exposure identified through the Colombian ZIKV disease surveillance system. Multidisciplinary clinical evaluations were provided to the twins during their first three years of life through a national program for children with in utero ZIKV exposure. Laboratory evidence of congenital infection as well as microcephaly, brain, eye, and neurodevelopmental compromise related to prenatal ZIKV infection were identified in only one infant of the twin pregnancy. This is the first report of monozygotic twins discordant for Zika-associated birth defects. The evaluation of the pathophysiology of discordance in disease outcome for congenital infections in twin pregnancies may lead to a better understanding of potential complex environmental and genetic interactions between the mother, her offspring, and an infectious exposure.

## 1. Introduction

Zika virus (ZIKV) is a single-stranded RNA virus that belongs to the *Flaviviridae* family. Human infection occurs from transmission by mosquito bites (*Aedes Stegomyia* species), sexual exposure, vertical transmission during the prenatal period, and blood transfusions [1]. Prenatal exposure to ZIKV is associated with diverse central nervous system (CNS) anomalies, which relate to the virus´ tropism for infection of intermediate neural progenitor cells and immature neurons, increasing cell death and altering cell cycle pathways to induce tissue damage in the developing CNS [2,3].

Prenatal ZIKV infection has been associated with structural birth defects of the brain and the eye and subsequent neurodevelopmental disabilities including epilepsy, hearing loss, body tone and movement abnormalities, and developmental delay, among others [4,5,6]. The prevalence of Zika-associated birth defects is between 5 and 14% following prenatal ZIKV infection [7]. Since the ZIKV outbreak in the Americas and the Caribbean in 2015–2016, academic research and public health surveillance programs have contributed to significant advancement of our understanding of the complex pathology and the spectrum of abnormal neurological and other clinical outcomes.

During the ZIKV epidemic in Colombia (October 2015 to June 2016), the Colombian National Institute of Health (INS) included ZIKV in its national surveillance system (SIVIGILA) [8,9]. Due to the urgency to understand the impact of in utero ZIKV exposure, INS monitored and evaluated a cohort of infants with prenatal ZIKV exposure from March 2017 to August 2019 through a special ZIKV surveillance program, including enhanced follow-up of pregnant women with ZIKV and their infants. The longitudinal evaluation of children in this program occurred over their first three years of life.

From this cohort, we describe a unique monozygotic (MZ) dichorionic-diamniotic (DCDA) twin pregnancy with discordant clinical outcomes in the twins. The analysis of ZIKV infection within a twin pregnancy can help inform our understanding of the diversity of outcomes and the impact of pregnancy-related factors such as placental defense mechanisms and genetic profiles as important pathophysiologic determinants of disease. This is the first report of MZ twins discordant for Zika-associated birth defects.

## 2. Materials and Methods

As part of the Colombian INS ZIKV surveillance program, multidisciplinary quarterly evaluations of infants from Zika-exposed pregnancies were planned and included (1) prenatal and postnatal clinical data abstraction of medical records provided by parents and/or caregivers, (2) ophthalmological evaluation of fundus employing indirect ophthalmoscopy with 30D lens and retinoscopy using a wide-field digital imaging system, after pupil dilation, (3) pediatric developmental and neurological assessments, and 4) evaluation by a clinical geneticist for a subset of children.

### 2.1. Anthropometric Evaluations

Fetal biometry including head circumference (HC), biparietal diameter (BPD), abdominal circumference (AC), and femur length were abstracted from serial obstetric ultrasonography studies. Gestational age (GA), estimated fetal weight and percentiles, and standard deviations (SD) of fetal measurements were calculated using INTERGROWTH 21st^®^ fetal growth standards [10,11]. At each postnatal visit, weight, length, and HC measurements were taken by a pediatrician. Percentiles and SD were calculated using INTERGROWTH 21st^®^ standards according to GA and sex for measurements taken between 0 and 14 postnatal days [12]. Percentiles and SD for subsequent measurements were calculated using the World Health Organization (WHO) growth standards according to sex and age [13]. Microcephaly was defined as an HC more than 2 SD below the mean for age and sex (or GA for birth measurements). Short stature was defined as length for age more than 2 SD below the mean, and underweight was defined as weight for age more than 2 SD below the mean.

### 2.2. Laboratory Testing

As part of SIVIGILA, serologic testing to identify congenital infections including cytomegalovirus, *Toxoplasma gondii*, rubella, herpes, syphilis, human immunodeficiency virus (HIV), and hepatitis B virus were performed on the mother and infant following national guidelines on clinical care of women with ZIKV infection during pregnancy and national guidelines on clinical approach to infants with birth defects possibly associated with ZIKV [14,15].

For all mothers who had symptoms of ZIKV infection during pregnancy, a serum sample was taken to test for ZIKV ribonucleic acid (RNA) and ZIKV immunoglobulin M (IgM) antibodies. If the infant was born with a CNS birth defect, a serum sample was requested for ZIKV testing. The presence of ZIKV RNA was analyzed using either a singleplex assay [16] or a Trioplex real-time reverse transcription-polymerase chain reaction (rRT-PCR) assay [17] depending on the availability of the test. ZIKV IgM antibodies were assessed in maternal or infant serum using Zika virus Detect TM IgM 1.0 Capture ELISA Kit (Inbios, Seattle, WA, USA). A plaque reduction neutralization test (PRNT) was performed on serum samples incubated at 56 °C for 30 min. Human labile serum factor was added back to the sample, and two-fold serial dilutions were incubated with ~100 plaque forming units of ZIKV for one hour at 37 °C, after which, the samples were plated in duplicate confluent monolayers of Vero cells within wells of a 6 well plate. Serum negative controls were run similarly, and a 90% neutralization threshold was determined in the plates compared to the serum negative control following CDC protocol. Titers were expressed as the reciprocal of the highest dilution in which ≥90% neutralization was observed.

Chromosome analysis (karyotyping at 450 bands) in whole blood was performed in infants with any birth defect as part of the surveillance protocol. Whole blood molecular analysis of polymorphic markers to identify allele concordance was performed to determine zygosity.

### 2.3. Ethics

The multidisciplinary clinical evaluations led by the INS were embedded within the national public health response to the ZIKV outbreak in Colombia. As such, they were considered as public health practice by INS per Colombian legislation [18], which is in compliance with the Declaration of Helsinki. At each evaluation, which was voluntary, parents or legal guardians were informed about the procedures involved during the visits and provided written consent when accepting their child´s participation. The use of clinical pictures and abstraction of clinical data considered relevant to the study was authorized by parents. The consent indicates that data provided could be used for academic purposes. Additionally, before submission of this article, a separate verbal consent was obtained from the parent for publication.

## 3. Case Report

### 3.1. Pregnancy Information

A 28-year-old gravida 4 parity 3 woman was evaluated from her first trimester of pregnancy in 2016. She resided in an area of Colombia with documented ZIKV transmission and had three previous uncomplicated pregnancies. She denied consanguinity with her male partner, and her family history was negative for congenital anomalies or genetic diseases. At 11 weeks of gestation, based on last menstrual period, an obstetric ultrasound detected a DCDA twin pregnancy with well-developed embryos and two separate placentas. During the first trimester of pregnancy, she was asymptomatic, but her male partner experienced fever, rash, arthralgia, and conjunctivitis; although there was clinical suspicion of ZIKV infection, no laboratory testing was performed on her or her partner. At 29 4/7 weeks of gestation, a routine obstetric ultrasound and Doppler examination showed discordant fetal growth, with an estimated fetal weight difference greater than 25% and normal umbilical artery waveform patterns. One fetus (Twin A) did not have growth compromise or any identified abnormalities. In the other fetus (Twin B), microcephaly was noted with HC and BPD measurements below the third percentiles for gestational age (Table 1). For this fetus, an increase in the size of the cisterna magna and diminution in the size of the cerebellum were also described. These findings were persistent during subsequent ultrasonography studies.

The mother remained asymptomatic during pregnancy. Maternal testing for congenital infectious agents (rubella, *Toxoplasma gondii*, cytomegalovirus, HIV, herpes, syphilis, and hepatitis B) was negative for recent infection, although maternal ZIKV testing was not performed. The twins were delivered at 37 1/7 weeks of gestation by scheduled cesarean section without maternal or neonatal complications. A PRNT analysis performed 11 months after the twins’ births demonstrated the presence of ZIKV antibodies in maternal serum.

### 3.2. Infant Description

#### 3.2.1. Twin A

Twin A, a male, had normal Apgar scores (8/10 at 1 min and 9/10 at 5 min) and adequate neonatal adaptation. The anthropometry at birth was HC of 34 cm (+ 0.74 SD, 77th percentile), weight 2330 g (−1.49 SD, 7th percentile), and length of 46 cm (−1.09 SD, 13th percentile). On physical examination, no morphological or abnormal phenotypic findings were described. He was admitted to the neonatal intensive care unit due to low birth weight, where he progressed favorably without complications. At 6 days of age, he had a normal cranial ultrasound. At 9 days of age, head computed tomography (CT) was performed due to neurologic concerns in his twin brother and showed a normal brain structure. At this same age, an ophthalmological examination of the fundus was within normal parameters for both eyes. He was discharged home at 11 days of age with adequate weight gain and without any indication of neurological involvement.

**Figure 1:**
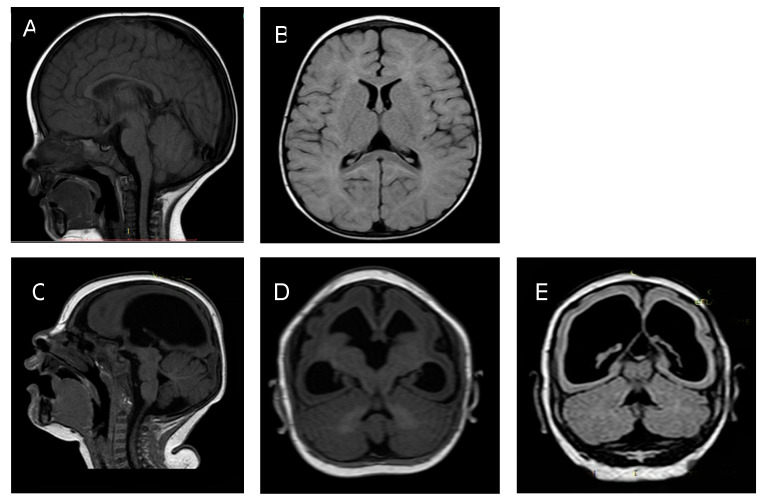
Twin A: Panel **A**: Brain MRI at 7 months of age sagittal T1 showing normal brain cortex formation and thickness, normal corpus callosum, and posterior fossa. Panel **B**: Axial T2 Fluid Attenuated Inversion Recovery (FLAIR) showing normal ventricular size. Twin B: Panel **C**: Brain MRI at 7 months sagittal T1. Panel **D** axial T2 FLAIR. Panel **E** axial T1. Images show a thin cortical mantle, ex vacuo enlargement of lateral ventricles, reduced cortical folds, and hypogenesis of the corpus callosum.

Subsequent routine medical evaluations reported normal development but altered growth with a diagnosis of short stature. Testing for infectious agents including *Toxoplasma gondii*, cytomegalovirus, rubella, and herpes virus taken at 4 and 7 months of age was negative for recent infection, and at 7 months, ZIKV IgM was negative. A PRNT analysis done at 11 months of age did not demonstrate the presence of ZIKV antibodies. He entered the INS ZIKV cohort at 6 months of age due to possible ZIKV prenatal exposure based on findings in his twin brother. At this time, his mother reported no health issues or specific concerns. At 7 months of age, a brain magnetic resonance imaging (MRI) study showed possible pituitary hypoplasia with no other structural compromise (Figure 1, panels A and B). At this time, ophthalmological evaluation and diagnostic auditory brainstem responses (ABR) were normal. Hormonal testing including thyroid hormone (TSH and free T4), somatomedin C, adrenocorticotropin (ACTH), and cortisol levels was within normal limits. Parental heights were within the expected ranges for the Colombian population.

Figure 1. Comparative brain imaging findings in monozygotic dichorionic-diamniotic twins discordant for congenital Zika virus outcomes.

**Figure 2:**
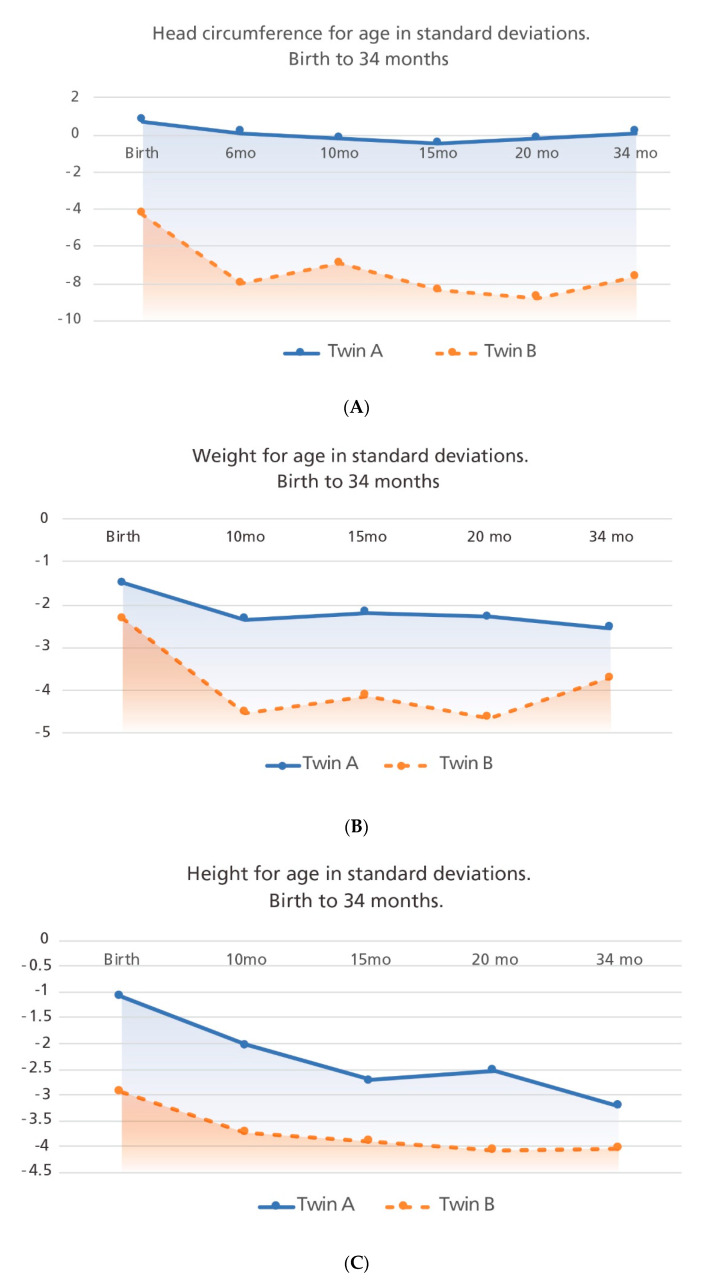
Growth trajectories for both twins taken at five time-points are shown. Panel **A**: head circumference for age, Panel **B**: weight for age and Panel **C**: height for age. Birth measurements were analyzed with Intergrowth 21st newborn growth standards comparing the measurement with gestational age (GA) and sex; other measurements were analyzed with the World Health Organization (WHO) growth standards comparing measurements with chronological age and sex.

**Figure 3:**
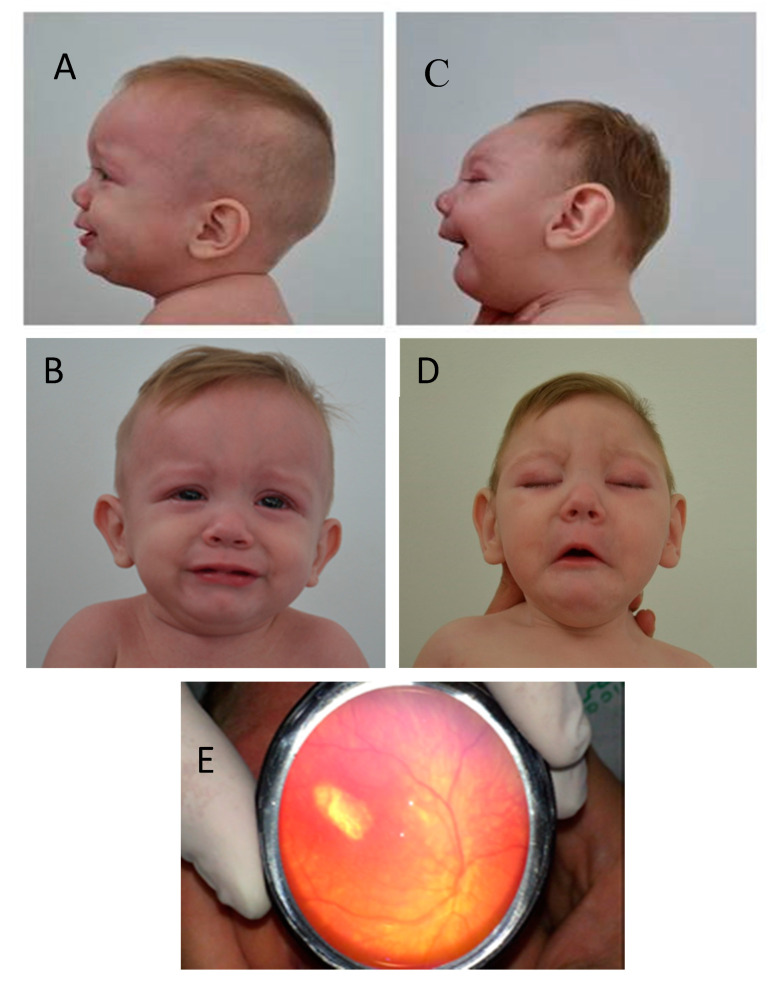
Twin A Panels **A** and **B**: Face and facial profile photographs of the head at 11 months of age show a normal head size and shape. Twin B Panels **C** and **D**: Face and facial profile of the head at 11 months of age show microcephaly, craniofacial disproportion, small cranial vault, sloped forehead, and micro-retrognathia. Panel **E** (Twin B): shows fundoscopic examination of the left eye revealing a hypopigmented macular scar. (Panel **E**, photograph courtesy of Pedro Acevedo, MD).

During his last evaluation, at 34 months of age, no specific medical issues were reported by his family. He had an HC of 49.5 cm (0.11 SD, 54th percentile), weight of 10.4 kg (−2.55 SD, <3rd percentile), and length of 84.1 cm (−3.22 SD, <3rd percentile). His growth trajectory over five visits is shown in Figure 2. His physical and neurological examinations showed no abnormal findings (Figure 3, panels A and B). He is currently under follow-up by a general pediatrician and a pediatric endocrinologist due to short stature, but no hormonal etiology has been identified. A molecular test of polymorphic markers was performed at 34 months of age on both twins showing identical genetic profiles, determining monozygosity.

Figure 2. Comparative infant growth trajectories in monozygotic dichorionic-diamniotic twins discordant for congenital Zika virus outcomes.

Figure 3. Comparative clinical characteristics of monozygotic dichorionic-diamniotic twins discordant for congenital Zika virus outcomes.

#### 3.2.2. Twin B

Twin B, a male, had normal Apgar scores (8/10 at 1 minute and 9/10 at 5 minutes) and adequate neonatal adaptation. The anthropometry at birth was HC of 27 cm (−4.28 SD, <3rd percentile), weight of 1960 g (−2.33 SD, <3rd percentile), and length of 42 cm (−2.94 SD, <3rd percentile). His physical examination revealed craniofacial disproportion due to a small cranial vault and microcephaly, nonpalpable anterior and posterior fontanelles, and overlapping parietal bones at the level of the sagittal suture. Neurological findings included a symmetric Moro reflex, positive suck reflex, normal muscle tone, and normal posture with symmetric flexion of the extremities, but he had a high pitch cry. No abnormal movements were described. Pulmonary, cardiac, abdominal, and skin examinations were normal. He was admitted to the neonatal intensive care unit due to low birth weight, microcephaly, and high metabolic and neurologic risk. At 1 day of age, a brain CT scan reported generalized cortical atrophy, scattered calcifications in the brain parenchyma, and supratentorial hydrocephalus.

Molecular and antibody analyses for ZIKV were performed at 5 days of age with a negative result for RNA detection but positive for ZIKV IgM. Testing for other congenital infections done at this same time including *Toxoplasma gondii*, cytomegalovirus, rubella, and herpes virus were negative, and his chromosomal analysis was normal. A PRNT analysis done at 11 months of age did not demonstrate the presence of ZIKV antibodies.

At 9 days of age, he was evaluated by a pediatric ophthalmologist who reported a normal right eye but an abnormal left eye with macular scarring, loss of retinal layers associated with changes of the pigmentary epithelium, and exposure of choroidal vessels and sclera but no signs of active intraocular inflammation. While in the neonatal intensive care unit, he remained without respiratory, cardiovascular, or neurological complications and was discharged home at 15 days of age.

At 2 months of age, his mother started to notice tremors. At 4 months of age, he had an electroencephalogram with a burst-suppression pattern, was diagnosed with epileptic encephalopathy by a pediatric neurologist, and was started on vigabatrin.

At 7 months of age, a brain MRI revealed severe microcephaly with thin cortical mantle, ex vacuo enlargement of lateral ventricles, reduced cortical folds, and hypoplasia of the corpus callosum (Figure 1 panels C, D, E). There were no calcifications noted on the brain MRI. Auditory evaluation by diagnostic ABR at this age remained normal. A second ophthalmological evaluation was unchanged from the neonatal assessment. At this same age, repeat serum testing for ZIKV IgM was negative.

During Twin B’s last evaluation at 34 months of age, his mother reported persistent epilepsy and abnormal movements consistent with tremors. He had swallowing difficulties but was able to be fed thickened liquids by mouth. His anthropometry showed an HC of 38.5 cm (−7.67 SD, < rd percentile), weight of 8.96 kg (−3.72 SD, <3rd percentile), and height of 81.1 cm (−4.04 SD, <3rd percentile), consistent with persistent severe microcephaly with a general compromise of somatic growth (Figure 2). On physical examination, he had microcephaly, craniofacial disproportion due to a small cranial vault, a non-palpable anterior fontanelle, a sloped forehead, straight palpebral fissures, anteverted nostrils, thin lips, and micro-retrognathia (Figure 3 panels C and D). His ears were low-set and posteriorly rotated; no other morphological abnormalities were documented. Neurological examination detected poor head control, with neck and truncal hypotonia and limb spasticity; he maintained an abnormal posture with hyperflexion of his upper extremities and hyperextension of his lower extremities. He had persistent suck and Moro reflexes and symmetrically brisk deep tendon reflexes. He had severe motor and language delay. The ophthalmological evaluation reported failure to fix and follow visual stimuli, and the indirect ophthalmoscopy reported signs of torpedo maculopathy and retinal scar of the left eye, unchanged from previous evaluations (Figure 3 panel E).

He is currently being managed by a multidisciplinary team, involving a pediatrician, a pediatric neurologist, and a pediatric ophthalmologist in conjunction with physical, occupational, and language therapies. His electroencephalogram at 34 months of age continues to show abnormal findings consistent with multifocal epileptic activity and hypsarrhythmia pattern, and he is being treated with vigabatrin, carbamazepine, and levetiracetam at adequate doses but without adequate seizure control.

## 4. Discussion

We report a unique case of discordant clinical childhood outcomes from an MZ DCDA twin pregnancy with probable prenatal ZIKV exposure. Fetal growth disparities were documented on the early third trimester ultrasounds showing low estimated fetal weight, small head size, and structural CNS abnormalities in Twin B, in contrast to a normal growing and well-developed Twin A. At birth and throughout the first 3 years of age, Twin A has demonstrated a normal neurodevelopmental phenotype, and after detailed clinical, imaging, and laboratory assessments, no Zika-associated birth defects or neurologic sequelae were found. However, he remained underweight with short stature at his last evaluation. No etiology has been identified for his growth deficiency, but isolated growth deficiency has not been associated with congenital ZIKV infection. In contrast, Twin B´s prenatal and postnatal assessments revealed multiple Zika-associated birth defects with structural brain abnormalities compromising the cerebral cortex, deep white matter, and ventricular systems and eye abnormalities mainly affecting retinal structures. His neurologic sequelae include epilepsy, swallowing difficulties, and severe neurodevelopmental delay. These myriad clinical findings have been previously described in the context of prenatal ZIKV infection [19,20,21], where extensive CNS tissue damage that follows in utero exposure to this agent acts as the main pathologic event, leading to severe structural and functional compromise [22].

Due to the high prevalence of ZIKV in this area of Colombia during the pregnancy and the birth defects identified in Twin B, there was a concern for congenital ZIKV infection despite a lack of maternal symptoms and prenatal testing. Twin B was positive for ZIKV IgM in the neonatal period but negative at 7 months of age; serology testing was not performed in Twin A until 7 months of age, at which point it was negative. The PRNT performed at 11 months of age demonstrated the presence of ZIKV antibodies in maternal serum but absent antibodies in serum of both twins. However, based on the entirety of laboratory findings, including negative testing for other congenital infections and normal karyotype, as well as characteristic clinical findings, including brain and eye defects and neurodevelopment disabilities, Twin B’s compromise was most likely attributable to prenatal ZIKV infection. Twin A´s ZIKV infection status is not fully known, and although IgM positivity might have persisted at 7 months of age if prenatal infection had occurred, serology testing for ZIKV infection has variability in assay sensitivity and specificity that could limit ZIKV IgM identification [23]. IgM antibodies appeared to wane over time in one study of the association of microcephaly and congenital ZIKV infection with 20 of 43 infants (47%) with microcephaly lacking evidence of a recent ZIKV infection at follow-up; the oldest infant with positive IgM was 5 months of age at testing [24]. The finding of negative PRNTs in both twins at 11 months is somewhat unexpected, however, knowledge regarding the performance of PRNTs for ZIKV in infants is limited.

Congenital infections in twin pregnancies have been documented with vertical transmission by several infectious agents including rubella, cytomegalovirus, *Toxoplasma gondii*, lymphocytic choriomeningitis virus, and most recently, Zika virus [25,26,27,28,29,30,31]. Outcomes of twin pregnancies affected by congenital infections vary with neither twin, one twin, or both twins having laboratory and/or clinical findings of the disease. Environmental and genetic factors influence exposure, infection, and illness severity. Multiple gestation pregnancies may represent a prime source to better understand how some of these factors influence outcome, since the gestations are developing in the same womb. A series of ZIKV affected twin pregnancies have been reported in the literature, some with concordant and others with discordant outcomes. Twin discordance seems to be a more common finding among dizygotic (DZ) pregnancies, and it can be seen at multiple levels including within clinical and laboratory findings between the twins, between the placentas or the portions of a single placenta, and between the infant and corresponding placenta [32].

Van der Linden and colleagues reported two cases of DZ twin pregnancies impacted by ZIKV where only one of the infants of the pair was affected, showing both birth defects and laboratory evidence compatible with congenital ZIKV infection. However, the basis for the zygosity determination in the like-sexed pair was not reported [33]. In a larger case series, Caires et al. reported nine twin pregnancies with ZIKV infection; seven pairs were DZ, with discordant clinical findings seen in six of them. Both MZ pregnancies included in this analysis had concordant clinical findings related to congenital ZIKV infection. A further analysis that included in vitro ZIKV infection of neural progenitor cells (NPCs) derived from human induced pluripotent stem cells from three pairs of discordant DZ twins identified a significant difference of NPC survival and viral replication being more severe in samples from the affected twin of each pair [34]. Most of the reported cases of MZ twin pregnancies with congenital ZIKV infection have concordant clinical outcomes, either having absence of adverse outcomes [32] or compromise in both fetuses/infants [34,35]. For DZ and MZ pregnancies with concordant fetal and infant outcomes, the extent of the clinical compromise can be variable, including differential findings regarding CNS and eye abnormalities and of neurodevelopmental sequelae such as epilepsy, developmental delay, and hearing loss [32,34,35].

Perhaps the most critical environmental factor determining the extent of the congenital infection is the barrier at the maternal–fetal interface, the placenta. However, the mechanisms by which organisms breach this barrier and how they might vary by gestational age are not well understood [36]. In twin pregnancies, the configuration of the placenta and associated membranes vary by zygosity and timing of cleavage in MZ twins [37], and secondary changes such as fusion of dichorionic placentas or vascular anastomoses are common [38]. In dichorionic twins that can be either MZ or DZ, differences in fetal environments have been postulated as a factor in discordance in phenotypes [39]. A second environmental factor is the quantity or the load of the infectious agent in the maternal circulation and the amniotic fluid [27].

Another factor to be considered for this specific twin pregnancy is the possible scenario of a sexual transmission of ZIKV, as the mother´s male partner reported symptoms compatible with ZIKV infection during the first trimester of her pregnancy. Sexual transmission of ZIKV has been well documented [40] as well as the occurrence of associated birth defects when infection occurs through this route [41]. Animal models studying ZIKV sexual transmission have reported fetal infection in the absence of maternal viremia, leading to the possibility that a more localized and ascending infection from the vagina may enter the uterine environment without a hematogenous spread [42]. This route of ZIKV transmission has not been thoroughly documented in a human pregnancy but could to some extent help explain the discordant clinical outcomes seen in this twin pregnancy [43]; however, the mother’s PRNT indicates infection at some point in time.

Genetic factors impacting risk include genotypes of the mother or the fetus that affect immunity or genotype of the organism that affects virulence. Recent work in singleton pregnancies has identified an association between a genetic polymorphism affecting type I interferon antiviral responses in Zika-infected mothers and congenital Zika syndrome (CZS) in their infants and an association between a polymorphism in the tumor necrosis factor-α gene in infants and CZS and severe microcephaly outcomes [44]. Work with DZ twins and cells derived from NPCs has shown significantly different gene expression signatures of neurodevelopmental genes between non-affected and CZS-affected twins [34]. Among MZ twins, the traditional concept of identical twins with identical genotypes has been modified to recognize that post-zygotic genetic, epigenetic, and environmental events modify the original zygotic genome [39,45]. Post-zygotic modification of cells that eventually transform into the placenta, resulting in genetic differences between MZ placentas, has also been documented [46]. Additionally, discordance for pathologic changes consistent with ZIKV infection was demonstrated in different portions of a monochorionic placenta of twins born to a mother with ZIKV infection during pregnancy [32]. Thus, both genetic and environmental factors could lead to discordant phenotypes in MZ twins. In most reports, congenital ZIKV infection phenotypes (laboratory and/or clinical) in members of MZ twin pairs have been concordant [28,34,35,47]. Discordant phenotypes among MZ twins with congenital rubella [25] and toxoplasmosis [28] have been reported, but not previously in congenital ZIKV infection. Exploring the pathophysiology of how congenital infections result in different clinical outcomes in twin pregnancies (both DZ and MZ) could help in understanding the interplay between the in utero environment and between the host and the infectious agent and how this influences the disease course and its outcomes.

## 5. Conclusions

This is the first report of MZ twins discordant for Zika-associated birth defects. Laboratory evidence of congenital infection was only available for the affected twin; thus, ZIKV infectious status for the unaffected twin is unknown. Factors explaining clinical discordance could relate to the type of placentation seen in this MZ twin pregnancy, where two separate placentas can act as efficient barriers to protect the fetus from maternal infections. Additionally, epigenetic and post-zygotic genetic modifications could lead to differences in host responses and defense mechanisms against this infectious agent. A possible ascending route of infection secondary to sexual ZIKV transmission leading to a more localized viral distribution could also contribute to the difference in clinical outcome in this twin pregnancy. Special attention to congenital infections in twin pregnancies is encouraged, providing adequate diagnosis for all fetuses and newborns and prompting research efforts that may aid in the understanding of complex environmental and genetic interactions.

## Figures and Tables

**Table 1:** Obstetric Ultrasound Findings in Monozygotic Dichorionic-Diamniotic Twins Discordant for Congenital Zika Virus Outcomes.

	Twin A					Twin B				
GA by LMP (w/d)	GA by Fetal Biometry (w/d)	EFW g (% for GA)	HC mm (% for GA)	BPD mm (% for GA)	Main Findings	GA by fetal biometry (w/d)	EFW g (% for GA)	HC mm (% for GA)	BPD mm (% for GA)	Main Findings
11/0	11/0	NA	NA	NA	DCDA twin pregnancy	11/0	NA	NA	NA	DCDA twin pregnancy
29/4	28/2	1255 (32.6)	277 (59.9)	74 (10.6)	Normal CNS structures, cisterna magna diameter, cerebellar diameter. Normal FD-US.	25/5	883 (<3)	207 (<3)	52 (<3)	IUGR, microcephaly enlarged cisterna magna, small cerebellum. Normal FD-US.
31/2	30/2	1586 (44)	285 (34)	78 (10)	Normal FD-US.	29/0	1215 (1)	225 (<3)	59 (<3)	IUGR, microcephaly Normal FD-US.
35/2	33/4	2300 (34)	309 (26.3)	86 (12)	Normal FD-US.	31/6	1930 (6)	258 (<3)	61 (<3)	IUGR, microcephaly Normal FD-US.

Abbreviations: BPD: biparietal diameter, CNS: central nervous system, DCDA: dichorionic-diamniotic, EFW: estimated fetal weight, FD-US: Fetal-doppler ultrasound, GA: gestational age, g: grams, HC: head circumference, IUGR: intrauterine growth restriction, LMP: last menstrual period, mm: millimeters, NA: not available, US: ultrasound, w/d: weeks/days. Percentiles and standard deviations of fetal measurements were calculated using the Intergrowth 21st fetal growth standards.
